# Characterization of Microbiota Composition and Presence of Selected Antibiotic Resistance Genes in Carriage Water of Ornamental Fish

**DOI:** 10.1371/journal.pone.0103865

**Published:** 2014-08-01

**Authors:** Lenka Gerzova, Petra Videnska, Marcela Faldynova, Karel Sedlar, Ivo Provaznik, Alois Cizek, Ivan Rychlik

**Affiliations:** 1 Veterinary Research Institute, Brno, Czech Republic; 2 Department of Biomedical Engineering, Brno University of Technology, Brno, Czech Republic; 3 University of Veterinary and Pharmaceutical Sciences, Brno, Czech Republic; Institut National de la Recherche Agronomique, France

## Abstract

International trade with ornamental fish is gradually recognized as an important source of a wide range of different antibiotic resistant bacteria. In this study we therefore characterized the prevalence of selected antibiotic resistance genes in the microbiota found in the carriage water of ornamental fish originating from 3 different continents. Real-time PCR quantification showed that the *sul1* gene was present in 11 out of 100 bacteria. *tet(A)* was present in 6 out of 100 bacteria and *strA*, *tet(G)*, *sul2* and *aadA* were present in 1–2 copies per 100 bacteria. Class I integrons were quite common in carriage water microbiota, however, pyrosequencing showed that only 12 different antibiotic gene cassettes were present in class I integrons. The microbiota characterized by pyrosequencing of the V3/V4 variable region of 16S rRNA genes consisted of *Proteobacteria* (48%), *Bacteroidetes* (29.5%), *Firmicutes* (17.8%), *Actinobacteria* (2.1%) and *Fusobacteria* (1.6%). Correlation analysis between antibiotic resistance gene prevalence and microbiota composition verified by bacterial culture showed that major reservoirs of *sul1 sul2, tet(A)*, *tet(B) tet(G)*, *cat*, *cml*, *bla*, *strA*, *aacA*, *aph* and *aadA* could be found among *Alpha-, Beta-* and *Gammaproteobacteria* with representatives of *Enterobacteriaceae, Pseudomonadaceae, Rhizobiaceae* and *Comamonadaceae* being those most positively associated with the tested antibiotic resistance genes.

## Introduction

International trade with ornamental fish is gradually being recognized as an important source of a wide range of different antibiotic resistant bacteria [1], [2] since antibiotics in ornamental fish breeding are used both for therapy and as prophylactic treatment before transport [3]. This results in frequent and efficient positive selection of antibiotic resistant bacterial clones which, during periods of antibiotic withdrawal, may serve as reservoir of antibiotic resistance genes. Once an antibiotic resistant clone has been selected by antibiotic use, conditions in ornamental fish rearing, i.e. ambient temperature and aquatic environment, allow for horizontal transfer of antibiotic resistance genes to other population members by genetic elements of varying mobility, e.g. conjugative plasmids, transposons or integrons [4], [5]. The mobility of elements transferring antibiotic resistance is not limited by species or genus and genetic elements with the same antibiotic resistance genes can be found across a broad range of different bacterial species [6], [7]. Although absolute volumes of carriage water are quite small, frequent use of antibiotics, international trade, close contact to humans and bacteria adapted to an aquatic environment may result in the long term survival of such bacteria in new ecological niches where they may serve as a source of new (combinations of) antibiotic resistance genes for ‘domestic’ microbiota.

One of the methods used traditionally for assessing antibiotic resistance in complex bacterial populations is bacterial culture on nutrient agars with and without antibiotics. However, this provides only information on the antibiotic resistance in bacterial species which can grow under the given conditions and what may introduce certain bias. This is why culture-independent techniques such as quantitative real-time PCR or next generation sequencing are therefore increasingly used for characterizing the prevalence of a particular gene in a given bacterial community [8]–[10]. In addition, these techniques also allow for taxonomic characterization of the bacterial community itself [11], [12] although they cannot differentiate between live and dead cells. In this study we therefore tested samples of ornamental fish carriage water originating from 3 different continents immediately after their import to the Czech Republic with the aim to assess the prevalence of selected antibiotic resistance genes and characterize the microbiota composition in these samples. Since initial data indicated an increased abundance of integrons, we also determined the integron gene cassettes present in the microbiota of ornamental fish carriage water in detail. Finally we predicted the association of particular taxa with antibiotic resistance and confirmed the predictions by culture and antibiotic resistance characterization of isolates from selected bacterial families.

## Results

### Quantitative real-time PCR

**Figure 1:**
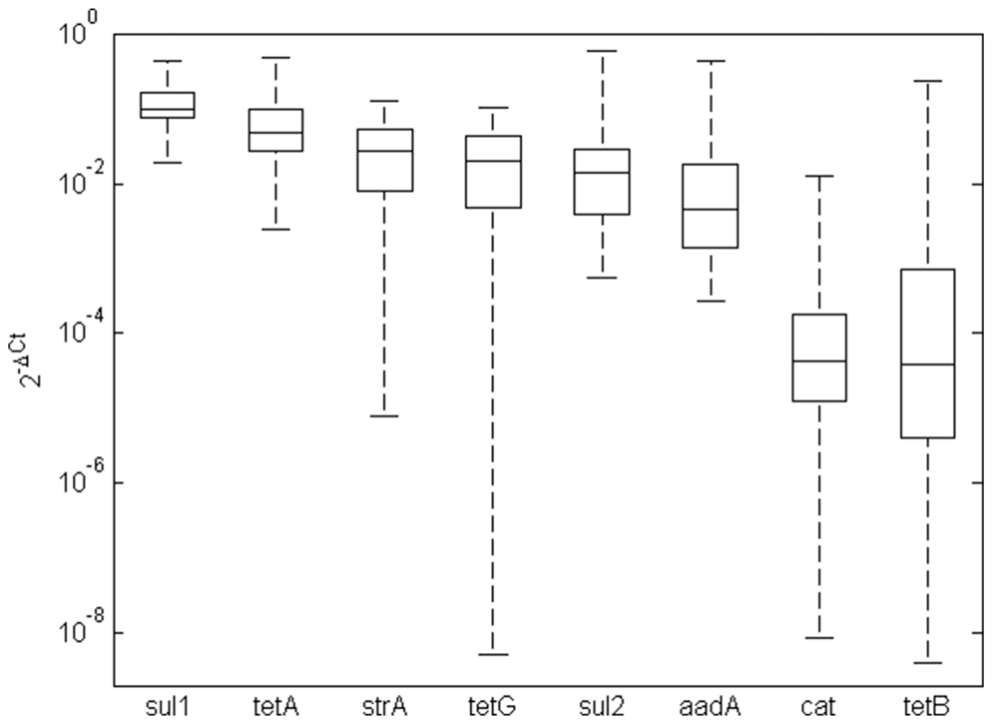
Prevalence of antibiotic resistance genes in ornamental fish carriage water. Antibiotic resistance gene prevalence is presented with the median, 25% and 75% percentiles (box) and the whiskers indicating the minimum and maximum values recorded.

*sul1* and *tet(A)* were the most prevalent genes out of those tested during initial sample screening. The median prevalence of *sul1* in microbiota of ornamental fish carriage water was 0.11 which corresponds to the presence of the *sul1* gene in 11 out of 100 bacteria (Fig. 1). The median prevalence of *tet(A)* in the microbiota of ornamental fish carriage water was 0.06 corresponding with the presence of *tet(A)* in approx. 6 out of 100 bacteria. The prevalence of *strA*, *tet(G)*, *sul2* and *aadA* was between 1–2 copies per 100 bacteria. The least prevalent were *cat* and *tet(B)* genes being present in less than 1 out of 10,000 bacteria. However, although *cat* and *tet(B)* were detected at a low prevalence on average, there were samples in which these genes were present at the same prevalence as the rest of the target genes (Fig. 1).

### Pyrosequencing of integron gene cassettes

**Figure 2:**
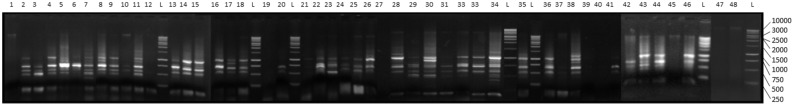
Amplification products after 5CS-3CS PCR of individual carriage water samples. L – molecular weight standard in bp. Amplification resulted in PCR products ranging from 200 to 1500 bp in size.

Since the *sul1* and *aadA* genes were present in ornamental fish water microbiota at a high prevalence (Fig. 1) and as both these genes are present in class I integrons [5], [7], we hypothesized that class I integrons must have been common in genomes of microbiota of ornamental fish carriage water. To address this hypothesis, we performed PCR with primers specific for 5′CS and 3′CS ends of class I integrons and this PCR resulted in positive amplification in 41 out of 48 samples (Fig. 2).

To identify gene cassettes present in class I integrons, the resulting PCR products were pooled and pyrosequenced. Pyrosequencing resulted into 223,877 reads longer than 200 bp. However, 188,201 reads were excluded by GS De Novo Assembler as containing repeated, chimeric or other corrupted sequences. Isotigs were therefore assembled from 18,806 reads and the remaining 16,870 reads represented singletons which, when submitted to GenBank, did not match any entry.

Altogether 33 different genes were detected in integron structures. Out of these, *qacE*, *dfrA22* and *aadA13* were the most frequent whereas *ereA*, *arr-8* or *qnrVC1* were detected only sporadically (Table 1). Only two integron gene cassettes with predicted functions, *nit2* and *estX*, were not associated with antibiotic resistance. *nit2* is a member of the nitrilase family which acts as a tumor suppressor in human cells [13] and *estX* encodes esterase X of unknown function found in integrons [14]. When the genes were grouped based on the type and mechanism of resistance, there were only 12 different antibiotic gene cassettes present in class I integrons (*qacE*, *dfr*, *aadA*, *aadB*, *aacA*, *aphA*, *cat*, *cmlA*, *blaP1*, *ereA*, *arr8* and *qnr*).

**Table 1:** Genes detected in class I integrons in carriage water microbiota of ornamental fish. Numbers show percentages out of a total of 18,806 reads.

Gene	*ΔqacE*	*dfrA22*	*dfrA1*	*dfrA12*	*dfrA5*	*dfrA21*	*dfrA27*	*dfr16*	*dhfrXVb*	*dfrA15*	*dfr2d*	*dfrA32*	*dfrB3*
**Antibiotic resistance**	QAC*	Tp	Tp	Tp	Tp	Tp	Tp	Tp	Tp	Tp	Tp	Tp	Tp
**Sequencing hits [%]**	41.64	18.13	4.47	1.81	0.29	0.17	0.15	0.09	0.08	0.02	0.01	0.01	0.01
**Gene**	*aadA13*	*aadA11*	*aadA1*	*aadA2*	*aadA15*	*aadA4*	*aadA3*	*aadB*	*aacA4*	*aacA3*			
**Antibiotic resistance**	Str	Str	Str	Str	Str	Str	Str	Str	AG	AG			
**Sequencing hits [%]**	10.75	2.26	1.66	1.37	0.19	0.12	0.02	0.01	1.55	0.52			
**Gene**	*cmlA1*	*cmlA2*	*catB3*	*blaP1*	*ereA*	*aphA15*	*arr-8*	*qnrVC1*	*nit2*	*estX*			
**Antibiotic resistance**	Cm	Cm	Cm	Amp	Ery	Kan	Rif	Enr	?	?			
**Sequencing hits [%]**	7.11	0.09	0.27	6.03	0.12	0.06	0.06	0.04	0.56	0.3			

*QAC-quaternary ammonium compounds, Tp-trimethoprim, Str-streptomycin, AG-aminoglycosides, Cm-chloramphenicol, Ery-erythromycin, Kan–kanamycin, Rif–rifampicin, Enr–enrofloxacin.

**Figure 3:**
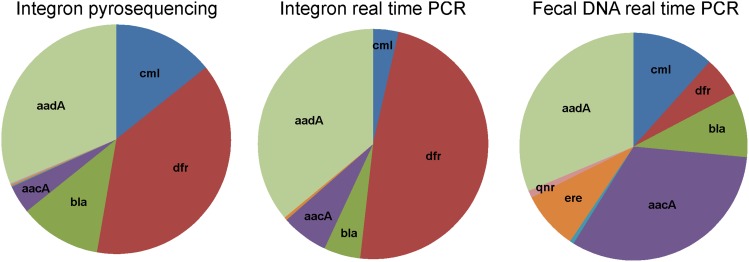
Distribution of selected genes in integron structures determined by pyrosequencing and real-time PCR. Average values calculated from data available for 48 samples were used for both real-time PCR based pie charts. Quantification of genes in integrons by pyrosequencing and real-time PCR resulted in similar results whereas the comparison of these results with the results from carriage water DNA indicated that *dfrA* was associated with integrons as its representation decreased when total carriage water was used as a template in real-time PCR. On the other hand, *aacA* and *ereA* must have been common in genetic elements different from integrons.

In the next step we verified the pyrosequencing results performed on the pooled sample by real-time PCR quantification of *aacA*, *ereA*, *dfrA22*, *cmlA1*, *blaP1*, *aphA*, *aadA*, *qnrVC1* and *arr-8* using individual samples. The real-time PCRs were performed with two different template DNAs, either with 5CS-3CS PCR products or with carriage water DNA. Real-time PCR using the former template allowed us to verify the results of pyrosequencing as the same template was subjected to the analysis. The results of real-time PCR using the latter template allowed us to predict which antibiotic resistance genes were dominantly associated with integrons and which were associated with genetic elements different from integrons. When average values were calculated from individual samples after real-time PCR using 5CS-3CS PCR products as templates, similar results to those obtained by pyrosequencing were recorded (Fig. 3). Performing real-time PCR with carriage water DNA as a template showed that *aacA* and *ereA* genes must have been commonly encoded by genetic elements different from integrons as their relative representation in total carriage water DNA was higher than in amplified 5CS-3CS PCR products. On the other hand, the lower representation of *dfrA* genes in carriage water DNA when compared with amplified 5CS-3CS PCR products indicated that this gene must have been common in integrons and was not commonly encoded by other genetic elements (Fig. 3).

### Microbiota composition determined by 16S rRNA pyrosequencing

Because of the high antibiotic resistance gene prevalence in the microbiota of ornamental fish carriage water, next we were interested in the microbiota composition in carriage water and predicting the bacterial taxa forming a reservoir of antibiotic resistance in such populations.

**Figure 4:**
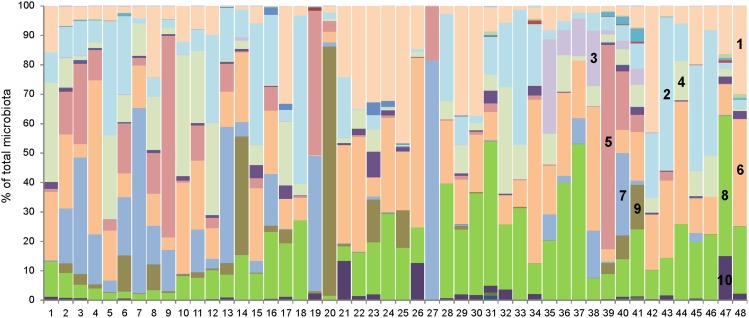
Composition of ornamental fish carriage water microbiota at class level. 1 – *Sphingobacteria*, 2 – *Gammaproteobacteria*, 3 – *Fusobacteria*, 4 – *Flavobacteria*, 5 – *Clostridia*, 6 – *Betaproteobacteria*, 7 – *Bacteroidia*, 8 – *Alphaproteobacteria*, 9 – *Bacilli*, 10 – *Actinobacteria*. For the full data set see table S1.

Pyrosequencing of 16S rRNA amplification products showed that carriage water microbiota were dominated by representatives of phylum *Proteobacteria* (48%), followed by *Bacteroidetes* (29.5%), *Firmicutes* (17.8%), *Actinobacteria* (2.1%) and *Fusobacteria* (1.6%). At a class level the dominating classes included *Betaproteobacteria* (20.7±17.2%), *Alphaproteobacteria* (14.5±7.2%), *Sphingobacteria* (12.2±8.5%), *Gammaproteobacteria* (11.7±6.9%), *Clostridia* (10.3±17.4%), *Bacteroidia* (9.7±9.8%) and *Flavobacteria* (7.6±5.3%) (Fig. 4 and table S1). However, individual ornamental fish carriage water samples were highly diverse and no similarities were observed in the microbiota composition according to country of origin or fish taxonomic species. In agreement with previous results, UniFrac analysis followed by PCoA also did not show up any separated cluster (data not shown).

**Figure 5:**
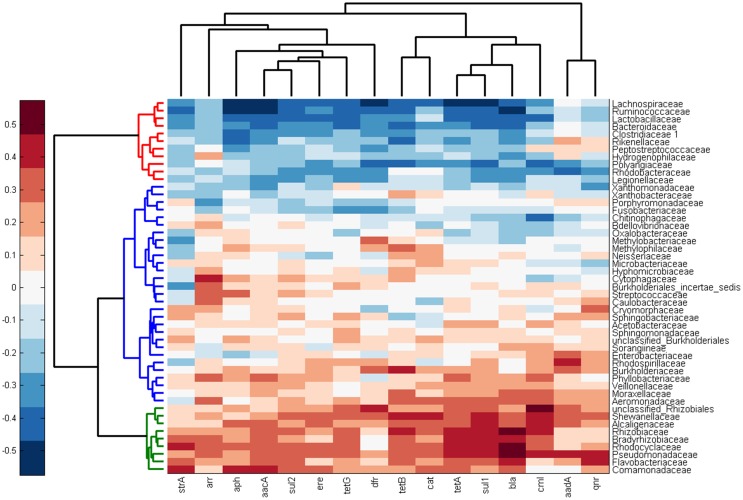
Heat map showing the correlation coefficients of the presence of individual families and particular antibiotic resistance genes. Three main clusters according to their positive and negative correlation with the prevalence of antibiotic resistance genes tested in this study are indicated by red, blue and green color.

Knowing the microbiota composition and antibiotic resistance gene prevalence for each of the 48 samples, in the next step we correlated these two parameters with each other. Into this analysis we included only the families which were present in more than a half of all samples, i.e. we excluded minority population members which provided false results when included. There were 3 main family groups according to their relationship to the prevalence of tested antibiotic resistance genes. The first group comprised 11 bacterial families which exhibited a negative correlation with the majority of antibiotic resistance genes quantified in this study (Fig. 5). Out of these, *Lachnospiraceae*, *Ruminococcaceae*, *Lactobacillaceae*, *Clostridiaceae* I, *Peptostreptococcaceae*, *Rickenellaceae* and *Bacteroidaceae* are common to the intestinal tract of warm-blooded animals and fish [11], [15]–[17] which may explain their origin in the carriage water.

The second cluster comprised of 29 bacterial families which exhibited a moderate positive correlation with the majority of antibiotic resistance genes tested in this study (Fig. 5). Representatives of *Enterobacteriaceae* and *Moraxellaceae* were clustered to this group.

The third group included 9 families which exhibited a high positive correlation with the majority of antibiotic resistance genes tested in this study. Representatives of *Pseudomonadaceae*, *Rhizobiaceae*, *Commamonadaceae* and *Flavobacteriaceae* were clustered to this group (Fig. 5).

In the last experiment we experimentally tested the predicted correlations in representatives of families *Pseudomonadaceae* (28 isolates), *Rhizobiaceae* (n = 4), *Flavobacteriaceae* (n = 8), *Comamonadaceae* (n = 22), *Enterobacteriaceae* (n = 17) and *Moraxellaceae* (n = 16). A new set of 42 carriage water samples had to be used for culture because the original samples were used only for DNA purification and not for bacterial culture. All the isolates were tested both for the presence of target genes and for the phenotypic expression of antibiotic resistance by disk diffusion assay.

**Figure 6:**
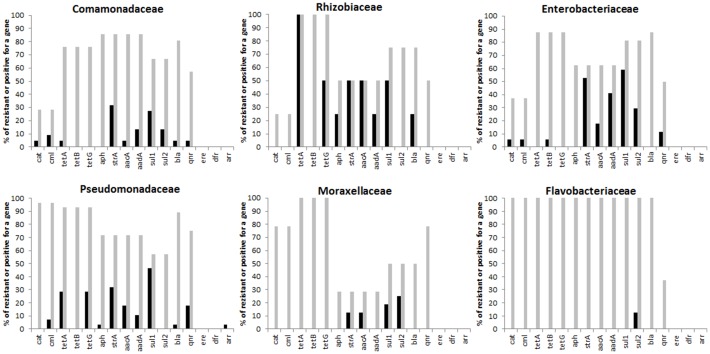
Antibiotic resistances in isolates belonging to families predicted as highly associated with antibiotic resistance in the microbiota of carriage water of ornamental fish. Data shows the percentage of isolates within a given family encoding the gene indicated on the X-axis out of all isolates tested. Black columns, results based on PCR detection of gene as indicated. Grey columns, percentage of antibiotic resistance determined in the same isolates by disk diffusion assay (ampicillin resistance is shown with *bla* gene, the same tetracycline resistance data are shown in *tet(A)*, *tet(B)* and *tet(G)* genes, streptomycin resistance is shown in *strA*, *aacA*, *aph* and *aadA* genes, chloramphenicol resistance is shown in *cml* and *cat* genes, sulphonamide resistance is shown in *sul1* and *sul2* genes and nalidixic acid resistance is shown in *qnr* gene. Resistance to erythromycin (*ere*), trimethoprim (*dfr*) and rifampicin (*arr*) was not determined by disk diffusion assay and therefore are not shown.

Antibiotic resistance genes targeted in this study were detected the most frequently in isolates belonging to the families *Pseudomonadaceae, Enterobacteriaceae* and *Rhizobiaceae*, although for *Rhizobiaceae* the conclusion was based only on 4 isolates. Isolates of families *Comamonadaceae* and *Moraxellaceae* encoded the tested antibiotic resistance genes the least frequently and with a single exception, none of the tested antibiotic resistance genes were confirmed in *Flavobacteriaceae* (Fig. 6). Except for *Flavobacteriaceae*, bacterial culture followed by gene detection confirmed the predictions based on the correlation of microbiota composition and antibiotic gene prevalence.

Antibiotic resistance tested in the same isolates by disk diffusion assay was extremely high. More than half of the isolates in all families were resistant to ampicillin, tetracycline, and sulphonamides. Resistance to tetracycline was the most common because between 75 to 100% of isolates in all 6 families were resistant to its activity. Isolates in families *Pseudomonadaceae* and *Flavobacteriaceae* exhibited the highest levels of antibiotic (multi)resistance out of the 6 families compared. In all families, but in *Flavobacteriaceae* in particular, it could be seen that the resistant phenotypes were only partially explained by the presence of tested genes and the resistances must have been caused genes different from those targeted in this study (Fig. 6).

## Discussion

The prevalence of *strA*, *sul1*, *sul2*, *tetA*, *tetG*, *aadA*, *aacA* and *cml* in microbiota of ornamental fish carriage water was very high and the *sul1* gene was present in more than 10% of all bacteria. This prevalence was 2–4 logs higher than in the feces of farm animals, which we characterized recently [10].

Despite a wide geographical distribution of sample origins, pyrosequencing of class I integron gene cassettes identified only a limited pool of genes. *ΔqacE*, *dfrA* and *aadA* genes were among those recorded most frequently, similar to previous findings [5], [7], [18]. On the other hand, we never recorded tetracycline resistance genes as part of integrons despite the fact that *tet(A)* and *tet(G)* genes were quite common in carriage water microbiota. The comparison of individual gene prevalence in total DNA and amplified integron sequences also showed that certain genes were common to class I integrons but were infrequently encoded outside of integrons. *dfrA22* coding for trimethoprim resistance is such an example as it was common in integrons but underrepresented in total DNA. On the other hand, the *aac* gene responsible for aminoglycoside resistance was a very common gene in the microbiota of ornamental fish but this gene was infrequently associated with integrons. This corresponds to studies of Hopkins et al. and Galimand et al. who showed that *aac* genes are a common part of transposons and conjugative plasmids separate from class I integrons [19], [20]. Interestingly, since class I integron gene cassettes were identified in this study without any bias and because these genes positively correlated with families belonging to *Alpha*-, *Beta*- and *Gammaproteobacteria*, this genetic element seems to be restricted to these classes belonging to phylum *Proteobacteria* and only rarely or not at all spread to bacterial species of phyla *Firmicutes* and *Bacteroidetes*.

Since an environment consisting of closed transport bags with ornamental fish was analyzed, the carriage water must have been influenced by fish gut microbiota. However, the composition of ornamental fish carriage water samples was similar to that of fresh water aquatic environments [12], [21], and mostly differed from fish gut microbiota [16], [22], [23], sea water microbiota, or even fecal material of warm blooded animals [11], [15], [17], [24], [25].

Correlation analysis between microbiota composition and prevalence of selected antibiotic resistance genes was performed to predict taxa likely serving as the most important antibiotic resistance gene reservoirs. When performing such an analysis, a certain number of limiting points must be taken into consideration. Firstly, even of the taxa harboring antibiotic resistance genes, not all isolates are resistant and absolute correlation in acquired antibiotic resistances cannot be expected. Secondly, different microbiota members may code for the same antibiotic resistance gene thus making the contribution of individual microbiota members rather difficult to determine. Thirdly, under-represented taxa may carry an antibiotic gene at a high frequency but this will unlikely cause a change in global antibiotic resistance gene prevalence and their positive correlation with a particular antibiotic resistance gene may remain unrecognized. Considering all these possibilities and also the fact that the predictions and bacterial culture verifications were performed in 2 different sets of samples, the fact that *tet(A)*, *tet(G)* or *sul1* genes were the most frequently identified in *Pseudomonadaceae* and *Rhizobiaceae* and less frequently in *Comamonadaceae* is in agreement with the correlation analysis. On the other hand, the widespread presence of *strA* among different taxa made predictions on its preferential association with a particular taxon less accurate. The correlation analysis also indicated that none of the families for which we collected isolates should be associated with *dfr* genes and in agreement, this gene was not detected in any single bacterial isolate. Finally, it would be mistaken to conclude that taxa which were identified in this study as negatively correlating with the tested genes represent an antibiotic sensitive part of a bacterial population. Though we did not prove this experimentally, results recorded for *Flavobacteriaceae* rather suggest that the majority of taxa forming carriage water microbiota are antibiotic resistant but due to the presences of genes different from those tested in this study.

In conclusion, the microbiota of carriage water from ornamental fish is commonly resistant to antibiotics. Moreover, since the same antibiotic resistance genes were present in bacteria belonging to different classes, ornamental fish breeding represents an ecological niche where horizontal gene transfer must be quite common. Predictions derived from culture-independent protocols allowed for the identification of the most frequent reservoirs of tested antibiotic resistance genes and together with culture-based confirmation indicated that horizontal gene transfer is limited to the class level and that the spread of the same genes among bacterial phyla is infrequent. Although absolute volumes of carriage water are quite small and acquired antibiotic resistance is small in scope when compared with intrinsic and natural resistances of certain bacterial species, ornamental fish breeding and trade represent an underestimated ecological niche with common presence of antibiotic resistance. The selection pressure then leads to selection of new combination of antibiotic resistance which can be transferred to domestic microbiota and/or to pathogens.

## Materials and Methods

### Ethics Statement

Owner of the company was aware that the samples were being collected for this study and gave their permission for doing so. Such permissions can be obtained from AC who, as a coauthor, was responsible for carriage water samples collection.

### Sample collection and DNA isolation

Forty-eight samples of ornamental fish carriage water were collected immediately after import to the Czech Republic. Countries of origin included Brazil (n = 1), China (n = 2), Hong Kong (n = 3), Indonesia (n = 10), Israel (n = 3), Nigeria (n = 1), Peru (n = 5), Singapore (n = 9), Thailand (n = 2) and Vietnam (n = 12). The bacteria were pelleted from the carriage water by centrifugation at 3000×g for 15 min at 4°C and the DNA was immediately extracted using QIAamp DNA Stool Mini kit following the manufacturer’s protocol (Qiagen). The quality and concentration of the purified DNA was determined spectrophotometrically and DNA was stored at −20°C until use.

### Quantitative real-time PCR

*sul1*, *sul2*, *strA*, *tet(A)*, *tet(B)*, *tet(G)*, *cat* and *aadA* genes were quantified by real-time PCR using primers listed in Table 2. Amplification of 16S rRNA genes was used as a reference to determine the total amount of bacterial DNA in each sample. PCR was performed in 3µl volumes in 384-well microplates using QuantiTect SYBR Green PCR Master Mix (Qiagen) and a Nanodrop pipeting station (Innovadyne) for dispensing PCR mixtures. PCR and signal detection was performed with a LightCycler II (Roche) with an initial denaturation at 95°C for 15 min followed by 40 cycles of denaturation at 95°C for 20 s, primer annealing at 61°C for 30 s and extension at 72°C for 30 s. Each sample was subjected to real-time PCR in triplicate and the mean values of the triplicates were used for subsequent analysis. The Ct values of genes of interest were normalized to an average Ct value of 16S rRNA gene amplification (ΔCt) and the relative amount of each gene of interest in given bacterial population was finally calculated as 2^−ΔCt^.

**Table 2:** List of primers used in this study.

Primer	Target	Primer sequence 5′-3′	Reference
strA_F	aminoglycoside phosphotransferase	ACCCTAAAACTCTTCAATGC	[10]
strA_R	aminoglycoside phosphotransferase	TCCCCAATACATTGAATAGG	[10]
sul1_F	dihydropteroate synthase	GTCTAAGAGCGGCGCAATAC	[10]
sul1_R	dihydropteroate synthase	GGATCAGACGTCGTGGATGT	[10]
sul2_F	dihydropteroate synthase	CGCAATGTGATCCATGATGT	[10]
sul2_R	dihydropteroate synthase	GCGAAATCATCTGCCAAACT	[10]
tetB_F	tetracycline efflux protein	TACAGGGATTATTGGTGAGC	[10]
tetB_R	tetracycline efflux protein	ACATGAAGGTCATCGATAGC	[10]
tetA_F	tetracycline efflux protein	CGATCTTCCAAGCGTTTGTT	[10]
tetA_R	tetracycline efflux protein	CCAGAAGAACGAAGCCAGTC	[10]
cat_F	chloramphenicol acetyl transferase	GGGAAATAGGCCAGGTTTTC	[10]
cat_R	chloramphenicol acetyl transferase	TCCATGAGCAAACTGAAACG	[10]
aadA_F	aminoglycoside adenyltransferase	CAGCCCGTCTTACTTGAAGC	[10]
aadA_R	aminoglycoside adenyltransferase	GATCTCGCCTTTCACAAAGC	[10]
tetG_F	tetracycline efflux protein	GATTGGTGAGGCTCGTTAGC	[10]
tetG_R	tetracycline efflux protein	GTGTTCCCGATTCTGTTGCT	[10]
aacA_F	aminoglycoside (6′)-acetyltransferase	CTGGGCAAAGGCTTGGGAAC	this study
aacA_R	aminoglycoside (6′)-acetyltransferase	AACCCCGCTTTCTCGTAGCA	this study
ereA_F	erythromycin esterase	CGCTCATTTTGTCGCGGAGT	this study
ereA_R	erythromycin esterase	GCACCGGCTGTTGAGTTGAG	this study
dfrA22_F	dihydrofolate reductase	ATGCACTGGCACTACCTCGT	this study
dfrA22_R	dihydrofolate reductase	GCGTCACCCTCGAAGGTTTG	this study
blaP-1_F	PSE-1/CARB-2 beta-lactamase	CAGGCTTCAATGGCAGAGCG	this study
blaP-1_R	PSE-1/CARB-2 beta-lactamase	AACAAGCGACGGAAAAGCCG	this study
aphA_F	aminoglycoside phosphotransferase	CGATCTCGCCTGACGATTGC	this study
aphA_R	aminoglycoside phosphotransferase	GCGTCGCCATGTGTGACTAC	this study
cmlA1_F	chloramphenicol efflux protein	CGCACAATAAGGCTCCTCGC	this study
cmlA1_R	chloramphenicol efflux protein	CTGCGGCTTACTTGTCTGCG	this study
qnrVC1_F	protein interacting with DNA/type-II-topoisomerases	GCTCGTGGCAACAGGAACAG	this study
qnrVC1_R	protein interacting with DNA/type-II-topoisomerases	AACCGCCAAACGTTGCGAAT	this study
arr-8_F	rifampin ADP-ribosylating transferase	AGGGTCGCACACTCAAGCA	this study
arr-8_R	rifampin ADP-ribosylating transferase	TATCCCCGGCCATCTAGACCA	this study
16Suniv_F	all bacteria	GAGGAAGGIGIGGAIGACGT	[30]
16Suniv_R	all bacteria	AGICCCGIGAACGTATTCAC	[30]
5CS	class I integron 5′ conserved sequence	GGCATCCAAGCAGCAAG	[28]
3CS	class I integron 3′ conserved sequence	AAGCAGACTTGACCTGA	[28]

### 16S rRNA gene PCR, pyrosequencing and data analysis

DNA samples from carriage water were individually amplified over the V3/V4 variable region of 16S rRNA [11]. PCR was performed using HotStarTaq *Plus* Master Mix Kit (Qiagen, Hilden, Germany) with initial denaturation at 95°C for 15 min followed by 30 cycles of denaturation at 94°C for 40 s, primer annealing at 55°C for 45 s and extension at 72°C for 1 min. The PCR products were separated on a 1.5% agarose gel and extracted from the gel with QIAquick Gel Extraction kit (Qiagen, Hilden, Germany). Purified PCR products were subjected to library preparation using the GS Amplicon Library Preparation Kit (Roche, Basel, Switzerland), emulsion PCR and pyrosequencing with the GS Junior Titanium kit strictly following the manufacturer’s instructions (Roche, Basel, Switzerland). The pyrosequencing was performed with the GS Junior 454 sequencer (Roche, Basel, Switzerland).

Data analysis was performed as described previously [11]. In brief, fasta and qual files generated as an output of the pyrosequencing were uploaded into Qiime software [26]. Quality trimming criteria included no mismatch in MID sequences and a maximum of 1 mismatch in primer sequences. The obtained sequences with a qual score higher than 20 were all shortened to a length of 350 bp and classified with RDP Seqmatch with an OTU discrimination level set to 97%. Diversity analyses on OTU clusters were performed both using all sequences available for each sample and using the same number of randomly selected sequences adjusted to the number of sequences available for the sample with the lowest coverage. Finally, UniFrac analysis [27] followed by principal coordinate analysis (PCoA) was used to characterize the diversity in the microbial populations tested.

### Integron PCR, pyrosequencing and data analysis

Gene cassettes present in integrons were PCR amplified using 5′CS and 3′CS primers [28] and *Taq* PCR Master Mix Kit (Qiagen, Hilden, Germany). The PCR cycling conditions consisted of initial denaturation at 94°C for 3 min followed by 35 cycles of denaturation at 92°C for 40 s, primer annealing at 55°C for 45 s and extension at 72°C for 3 min. The presence of amplification products was verified by electrophoresis on a 1.2% agarose gel. Equal volumes of amplification products from each sample were pooled together and subjected to library preparation using the GS Rapid Library Preparation Kit (Roche, Basel, Switzerland), emulsion PCR and pyrosequencing with the GS Junior Titanium series kits strictly following the manufacturer’s instructions (Roche, Basel, Switzerland). The ssf file generated as a sequencing output was used with De Novo Assembler software provided with the GS Junior to assemble the integron cassette sequences. Out of this analysis, the 454Isotig.fna file containing sequences of all gene cassettes present in integrons was uploaded into Blast2GO software to associate each isotig, i.e. transcript, with a gene designation according to the GeneBank [29]. In the next step, each isotig was associated with reads used for its assembly. The integron pyrosequencing results were finally verified by real-time PCR specific for the most frequent genes found in the integrons. Primer3 software was used for design of primers specific for *aacA*, *ereA*, *dfrA22*, *blaP1*, *cmlA1*, *aphA*, *qacEΔ*, *qnrVC1* and *arr-8* (Table 2). Quantitative real-time PCR using SybrGreen format was performed as described above.

### Verification of predicted associations of particular bacterial families and antibiotic resistance genes

To verify the predicted associations of particular bacterial families and antibiotic resistance genes, an additional 42 carriage water samples (i.e. in addition to 48 samples mentioned above) were collected and used for culture on Columbia blood agar, McConkey agar, Anacker and Ordal agar without any antibiotics at 25°C for 48–72 hours. Five morphologically different colonies were picked from each plate for species identification using MALDI Biotyper (Bruker Daltronik, Bremen, Germany), as recommended by the manufacturer. Phenotypic resistance to ampicillin, chloramphenicol, nalidixic acid, streptomycin, sulfonamides and tetracycline was tested by the disk diffusion method. Since interpretative criteria have not been defined for fish bacteria, the evaluation of results was based only on the presence/absence of inhibition zones.

DNA from 95 successfully identified colonies was extracted by boiling for 15 min and used as DNA template in real-time PCR with primers listed in Table 2. Real time PCR was used to maintain the same conditions, e.g. primer pairs or PCR mix, as in all previous PCR. In this case, real time PCR was not used for any quantification. PCRs in which the Ct values of the antibiotic resistance gene differed from the Ct value of 16S rRNA gene by less than 4 cycles and the melting temperature after the denaturation step following PCR was within 0.5°C of the expected range were considered positive whilst all other results were considered negative. The rather high 4 cycle difference in Ct values was used considering the different copy numbers of rRNA genes in different species as well as the potential presence of antibiotic resistance genes in (multicopy) plasmids.

### Statistics

Pyrosequencing results from 16S rRNA gene sequencing were analyzed by UniFrac analysis followed by principal coordinate analysis (PCoA) present in Qiime software. The Spearman’s rank correlation of microbiota with the prevalence of particular antibiotic resistance genes was visualized as a cluster heatmap using Ward’s method. The analysis was performed with MATLAB version 2013a (MathWorks, Natick, MA, USA).

## Supporting Information

Table S1 **List of all OTUs identified in this study.**
(XLS)
